# Elabela may regulate SIRT3‐mediated inhibition of oxidative stress through Foxo3a deacetylation preventing diabetic‐induced myocardial injury

**DOI:** 10.1111/jcmm.16052

**Published:** 2020-11-26

**Authors:** Cheng Li, Xiao Miao, Shudong Wang, Yucheng Liu, Jian Sun, Quan Liu, Lu Cai, Yonggang Wang

**Affiliations:** ^1^ Department of Cardiovascular Center The First Hospital of Jilin University Changchun, Jilin China; ^2^ The Second Hospital of Jilin University Changchun, Jilin China; ^3^ Osteopathic Medicine Candidate A.T. Still University School of Osteopathic Medicine in Arizona Mesa AZ USA; ^4^ Departments of Pediatrics Pediatric Research Institute The University of Louisville School of Medicine Louisville KY USA; ^5^ Norton Children Hospital Louisville KY USA

**Keywords:** apoptosis, diabetic cardiomyopathy, elabela, oxidative stress, SIRT3

## Abstract

Diabetic cardiomyopathy—pathophysiological heart remodelling and dysfunction that occurs in absence of coronary artery disease, hypertension and/or valvular heart disease—is a common diabetic complication. Elabela, a new peptide that acts via Apelin receptor, has similar functions as Apelin, providing beneficial effects on body fluid homeostasis, cardiovascular health and renal insufficiency, as well as potentially beneficial effects on metabolism and diabetes. In this study, Elabela treatment was found to have profound protective effects against diabetes‐induced cardiac oxidative stress, inflammation, fibrosis and apoptosis; these protective effects may depend heavily upon SIRT3‐mediated Foxo3a deacetylation. Our findings provide evidence that Elabela has cardioprotective effects for the first time in the diabetic model.

## 1 INTRODUCTION

Diabetes, a metabolic disease characterized by hyperglycaemia, is known to be caused by insulin resistance and insulin secretion dysfunction. In recent years, diabetes mellitus has become an epidemic and now represents one of the most prevalent disorders. The major cause of mortality and morbidity in diabetic patients is due to cardiovascular complications.^1^ Diabetic cardiomyopathy (DCM) is characterized, in its early stages, by diastolic relaxation abnormalities and, in later stages by clinical heart failure in the absence of dyslipidemia, hypertension and/or coronary artery disease.^2^ Persistent abnormal blood glucose induces oxidative stress, which promotes inflammation, myocardial interstitial fibrosis, and apoptosis. These abnormalities lead to cardiac remodelling and early diastolic and late systemic dysfunction.

The Apelin receptor, also called APJ or angiotensin receptor‐like 1, was first cloned in 1993 because of its strong sequence homology to the angiotensin II receptor. However, angiotensin II does not actually bind to APJ.^3^ The G‐protein‐coupled receptor APJ and its endogenous ligand Apelin are widely expressed in many tissue types including adipose, skeletal muscle and neuronal tissues of the hypothalamus. Apelin‐APJ signalling system is an important regulator of numerous physiological functions such as angiogenesis, cardiovascular function, fluid homeostasis, metabolic regulation, cellular proliferation/migration, apoptosis, oxidative stress and inflammation.^4^, ^5^, ^6^ Recently, more studies have revealed that APJ system is notably implicated in the development of various pathologies such as diabetes and its complications.^7^, ^8^, ^9^

Elabela, another endogenous ligand of APJ, has been identified as peptides produced and secreted by placenta,^10^ adult prostate, and kidney.^11^ Only in recent years has research successively revealed that Elabela and Apelin have similar functions, and both act via APJ to constitute the basic signalling axis of physiological processes.^12^ Previous studies showed that Elabela have protective effect on diabetic complications.^13^ In addition, treatment of Fc‐ELA‐21 fusion protein significantly increased angiogenesis, promoted cardiomyocyte proliferation and reduced apoptosis and heart fibrosis near the infarct area. But there is limit research about the Elabela effect on DCM.

Sirtuins are a family of nicotinamide adenine dinucleotide‐dependent histone deacetylases. They have been demonstrated to be key regulators of many cellular functions like metabolism, cellular growth and apoptosis.^14^ Within this family, SIRT3 is the main mitochondrial sirtuin that maintains metabolic and redox balance under physiological and pathological conditions. It is primarily found in the mitochondria of organs experiencing high metabolic activities, including hepatocytes, adipose tissue and cardiomyocytes.^15^ SIRT3 also participates in regulating the development of diabetic cardiac dysfunction.^16^, ^17^ A downstream regulator of APJ, SIRT3 has been shown to protect diabetic cardiomyocytes from oxidative stress‐mediated cellular injury.^18^, ^19^ This process may be related to deacetylation and activation of Forkhead box O3a (Foxo3a), thus up‐regulating antioxidative protein expression.^20^

In this study, we explored the functions of Elabela in DCM and the potential mechanism of Elabela that's linked to the development of DCM. Furthermore, we hypothesized that the protective effects of Elabela in DCM are mediated by its interaction with APJ and activation of SIRT3‐mediated Foxo3a deacetylation.

## 2 METHODS

### 2.1 Animal experiments

The animal protocols were approved by the Institutional Animal Care and Use Committee of the Jilin University and the animal studies were performed at the Animal Center of Jilin University. 6 weeks old, male C57BL/6J mice were purchased from the Beijing Wei Tong Li Hua Experimental Animal Technical Company (China) and housed in the Animal Center of Jilin University. All mice were housed in a room kept at 24℃ with 12:12 hour light/dark cycle. The type I diabetes model mice were established by intraperitoneally injecting with streptozotocin (STZ) (150 mg/kg).^21^, ^22^ Three days after injection, mice with blood glucose levels ≥ 250 mg/dL were considered diabetic. Elabela peptide (>98% purity) was purchased from GenScript (Nanjing, China), and was dissolved in phosphate buffered saline (PBS). The dose, timing and method of administration of Elabela were referred to our previous study.^13^

All animals were randomized into groups (8 per group) and raised for 3 months. The groups were labelled as follows: (a) Ctrl group: normal C57BL/6J mice treat with PBS; (b) Ctrl + Ela group: normal C57BL/6J mice treated with Elabela (4.5 mg/kg twice a day for 3 months, subcutaneous injection); (c) DM group: type 1 diabetic C57BL/6J mice treat with PBS; (d) DM + Ela group: type 1 diabetic C57BL/6J mice treated with Elabela (4.5 mg/kg twice a day for 3 months, subcutaneous injection). The blood glucose, bodyweight, and heart weight to tibia length ratio were measured at the end of the study.

### 2.2 Heart function measurement

Under anaesthesia, a small animal echo system (Vevo 770, Visual Sonics, Canada) with a high‐frequency ultrasound probe (RMV‐707B) was used to assess the mice cardiac function. We used pentobarbital (40 mg/kg) intraperitoneal injection anaesthetized mice. Left ventricular (LV) ejection fraction (EF), LV fractional shortening (FS), LV end‐systolic diameter (LVID, s), LV end‐diastolic diameter (LVID, d), heart rate, and LV end‐diastolic posterior wall thickness (LVPWd) were calculated before animals were killed.

### 2.3 Histology

Heart samples were fixed in 4% paraformaldehyde, paraffin‐embedded, and cut into 4‐μm‐thick sections. The sections were deparaffinized and stained with haematoxylin/eosin and Masson's trichrome to determine morphology and myocardial fibrosis respectively.

### 2.4 Real‐time Quantitative PCR

Total RNA was extracted from heart tissues using the AxyPrep™ multisource total RNA kit (Axygen Scientific, Inc). Then RNA was reverse transcribed to cDNA using the TransScript All‐in‐One first‐strand cDNA synthesis SuperMix (Transgen Biotech, Inc, Beijing, China). Real‐time PCR was performed using the TransStart Top Green qPCR SuperMix (Transgen Biotech, Inc, Beijing, China) and analysed by the ABI 7300 Real‐Time qPCR system. The primers of SIRT3 were purchased from Sangon Biotech (Shanghai, China), and the sequences were as follows: forward, 5′‐CGGCTCTACACGCAGAACATC‐3′ and reverse, 5′‐CAGCGGCTCCCCAAAGAA CAC‐3′.

### 2.5 Western Blot Analysis

The heart samples were homogenized in lysis buffer. After cryogenic centrifuge, the supernatant was collected. The protein concentration was measured by the Enhanced BCA Protein Assay Kit (Beyotime, Inc, China). Samples were diluted in loading buffer, heated at 95℃ for 5 minutes, separated by SDS‐PAGE, and transferred to PVDF membrane. Each membrane was pre‐incubated in tris‐buffered saline (TBS) containing 5% nonfat milk for 1 hour at room temperature and then incubated with specific primary antibodies (Table 1) overnight at 4℃. After washing in TBS with Tween 20, the membranes were incubated with secondary antibodies (Table 2) conjugated to horseradish peroxidase, and immunoreactive bands were visualized using the enhanced chemiluminescence reagent (Immobilon ECL Ultra Western HRP Substrate, Millipore Sigma, Inc, USA). Image J was used to analyse protein band density.

**Table 1:** The information of primary antibodies

Primary antibodies	Manufacturers	Source	Concentration
Bax	Abcam	Ribbit	1:1000
Bcl‐2	Abcam	Ribbit	1:1000
Caspase‐3	Abcam	Ribbit	1:800
Cleaved caspase‐3	Abcam	Ribbit	1:500
MCP‐1	Cell signaling	Ribbit	1:1000
MnSOD	Abcam	Ribbit	1:800
SIRT3	Abcam	Ribbit	1:1000
SOD‐2	Cell signaling	Ribbit	1:1000
TNF‐α	Cell signaling	Ribbit	1:1001
4‐HNE	Abcam	Ribbit	1:2000
β‐acin	Abcam	Mouse	1:5000

**Table 2:** The information of secondary antibodies

Secondary antibody	Manufacturers	Source	Concentration
Goat anti‐mouse IgG H&L	Beyotime (Inc,China)	Goat	1:1000
Goat anti‐Rabbit IgG H&L	Beyotime (Inc,China)	Goat	1:1000

### 2.6 Assessment of lipid peroxidation

Lipid peroxidation was estimated by measuring the peroxidation products malondialdehyde (MDA) and 4‐hydroxynonenal‐His adducts (4‐HNE). MDA was measured in homogenized heart tissues by commercially available kit (Nanjing Jiancheng Bioengineering Institute, China). MDA levels were expressed in nanomoles per milligram tissue. 4‐HNE was measured by Western blot.

### 2.7 TUNEL assay

Samples were fixed with formaldehyde, and antigen retrieval was performed by heat‐mediated citrate buffer, pH 6. Then, the tissues were incubated with 0.5% Triton X‐100 for 15 minutes at room temperature, and 1% BSA for 30 minutes at 37℃. Sarcomeric α‐actinin antibody was used at 1/200 dilution to incubate sample with for 16 hours at 4°C. Secondary antibody was used at 1:350 (coloured red). Then, TUNEL staining kit (DeadEnd™ Colorimetric TUNEL System, Promega, Inc, USA) was used to measure apoptosis (coloured green). Paraffin sections were dewaxed and incubated with TdT and fluorescein‐labelled dUTP for 60 minutes at 37℃. Nuclei were stained with DAPI (coloured blue). Images were acquired using confocal microscopy. Results were analysed using ImageJ. All assays and analyses were performed in a blind manner.

### 2.8 Immunoprecipitation

Protein extracts were prepared using the Extraction Reagents kit (Thermo Fisher Scientific, Basingstoke, UK). Two milligrams of protein from samples was used for immunoprecipitation with a Pierce^®^ Crosslink IP kit (Pierce) following the manufacturer's protocol and analysed by Western blot.

### 2.9 Statistical analysis

Data are presented as means ± SD for each group. Comparisons among groups were performed using one‐way ANOVA with post hoc pairwise repetitive comparisons using Tukey test with Origin 8.0 Lab data analysis and GraphPad Prism 6.0 graphing software. Statistical significance was considered as *P* < 0.05.

## 3 RESULTS

### 3.1 General features of DM mice with or without Elabela treatment

**Figure 1:**
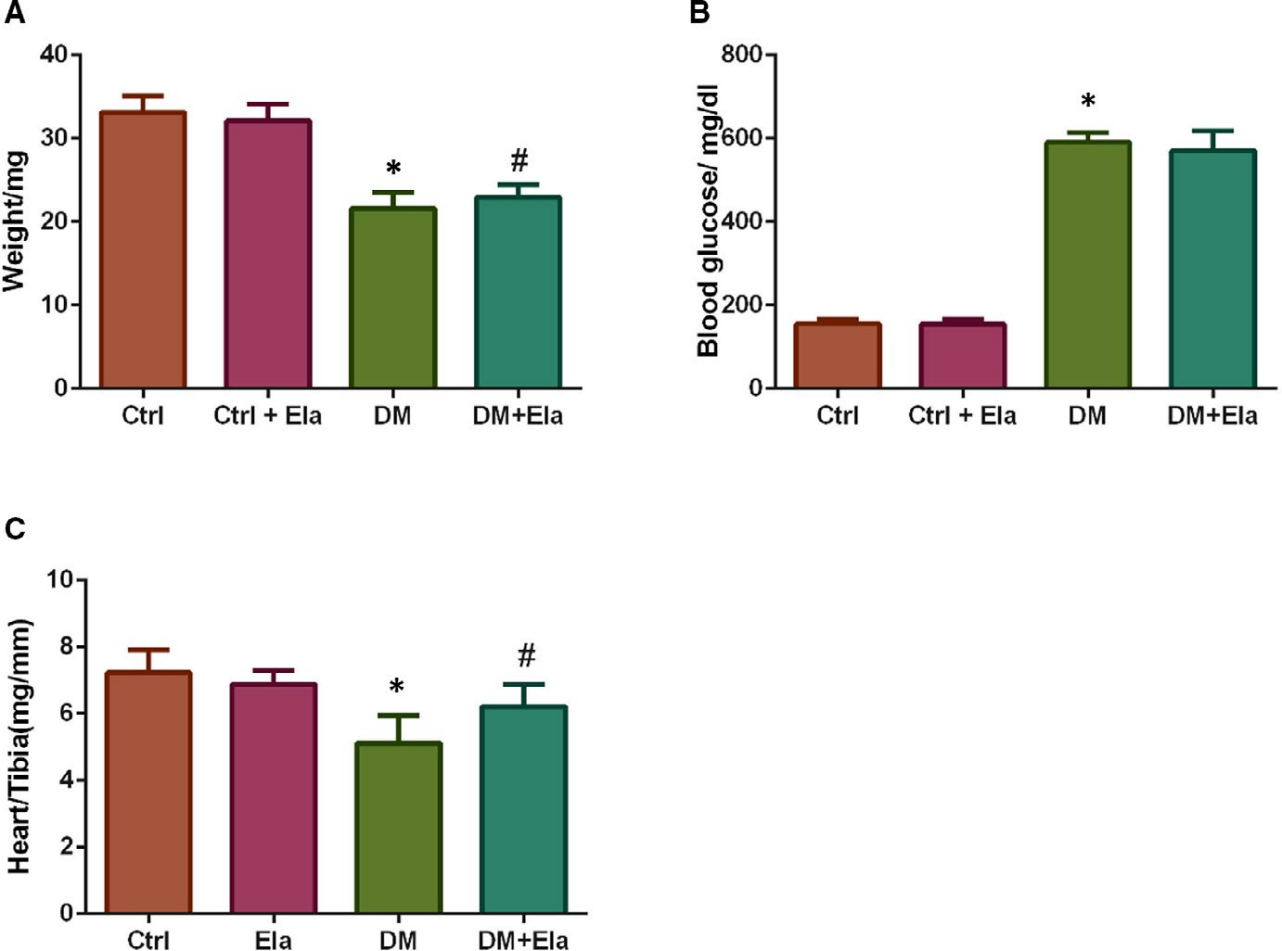
Bodyweight, blood glucose and heart weight to tibia length ratios were measured at the end of the study of different group. n = 8. *P < 0.05 DM group vs Ctrl group, ^#^ *P* < 0.05 DM + Ela group vs DM group. DM, diabetes mellitus

The bodyweight, blood glucose and the heart weight to tibia length ratio of DM and Ctrl mice were measured after treatment with or without Elabela for 3 months (Figure 1). After 3 months, the data showed that the bodyweight of the DM group has significantly decreased when compared to the Ctrl group. The DM + Ela group's bodyweight was slightly above the DM group (Figure 1A). However, the DM + Ela group's and the DM group's blood glucose levels were found to be relatively similar (Figure 1B). Meanwhile, we also measured the heart weights of each group, and calculated the heart weight to tibia length ratios (Figure 1C). The results showed that the heart weight to tibia length ratio was lower in diabetic mice compared to the control mice, but the DM + Ela group has slightly increased ratio compared to the DM group.

### 3.2 Effect of Elabela on diabetic cardiac function

**Figure 2:**
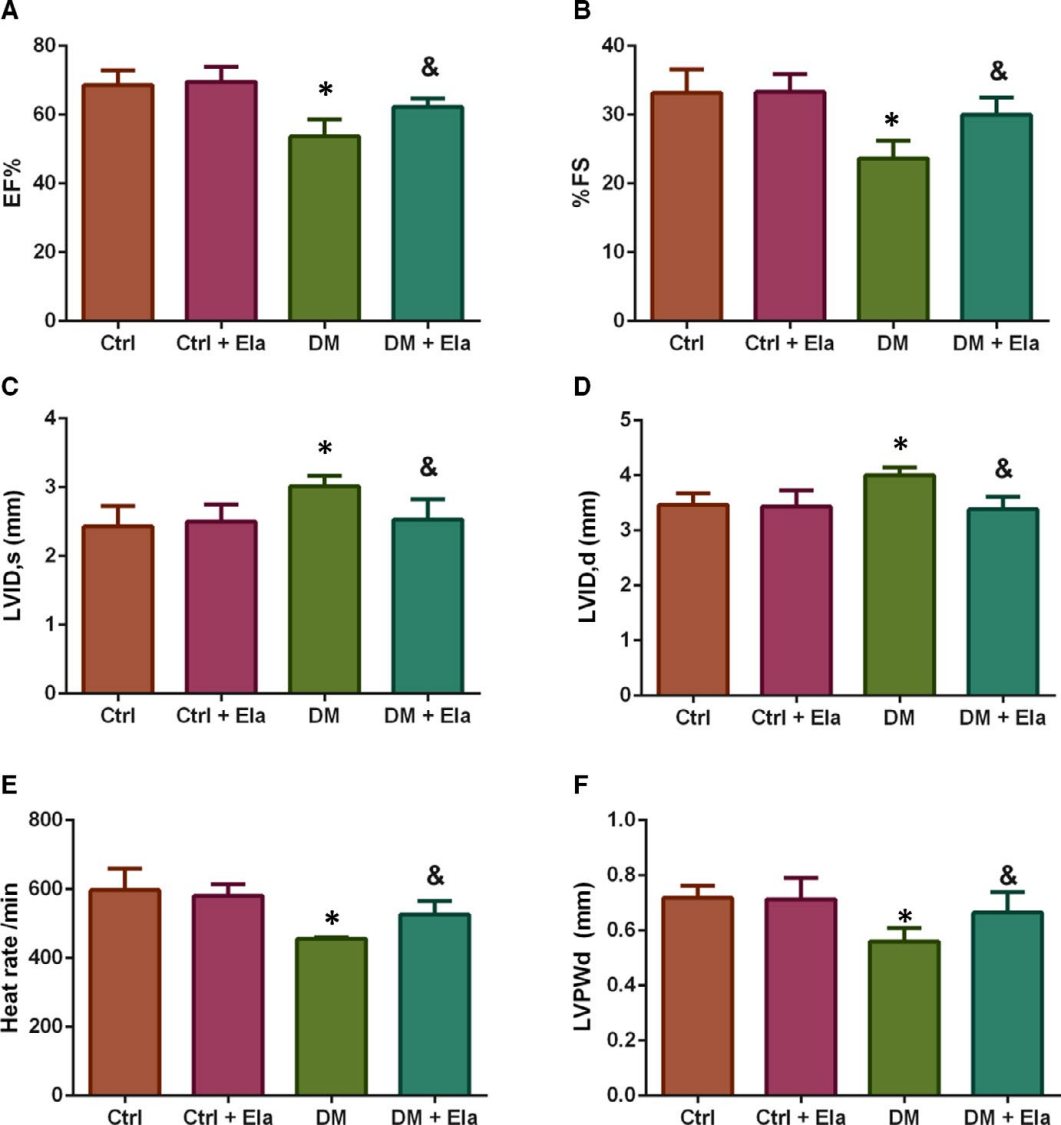
Cardiac function of different groups was evaluated by transthoracic echocardiography. n = 8. *P < 0.05 DM group vs Ctrl group, ^&^ *P* < 0.05 DM + Ela group vs DM group. DM, diabetes mellitus

We also used the transthoracic echocardiography system to evaluated cardiac function (Figure 2). We selected EF and FS as indicators in the evaluation of cardiac systolic function. We found that the DM group have lower EF and FS, compared to the Ctrl group; while diabetic mice treated with Elabela had significantly increased EF and FS compared to the DM control group (Figure 2A,B). Similarly, we selected LVID, s and LVID, d to evaluate cardiac systolic and diastolic function. The results showed that the DM group have larger LVID, s and LVID, d than the Ctrl group; moreover, the DM + Ela group's the LVID, s and LVID, d are significantly less than the DM group (Figure 2C,D). In addition, we selected heart rate and LVPWd as hallmark of myocardial remodelling (Figure 2E,F). The results showed that the DM group have lower heart rate and LVPWd than the Ctrl mice; the DM + Ela group's heart rate and LVPWd are significantly higher than the DM group.

### 3.3 Effect of Elabela on diabetes‐induced cardiac pathological changes

**Figure 3:**
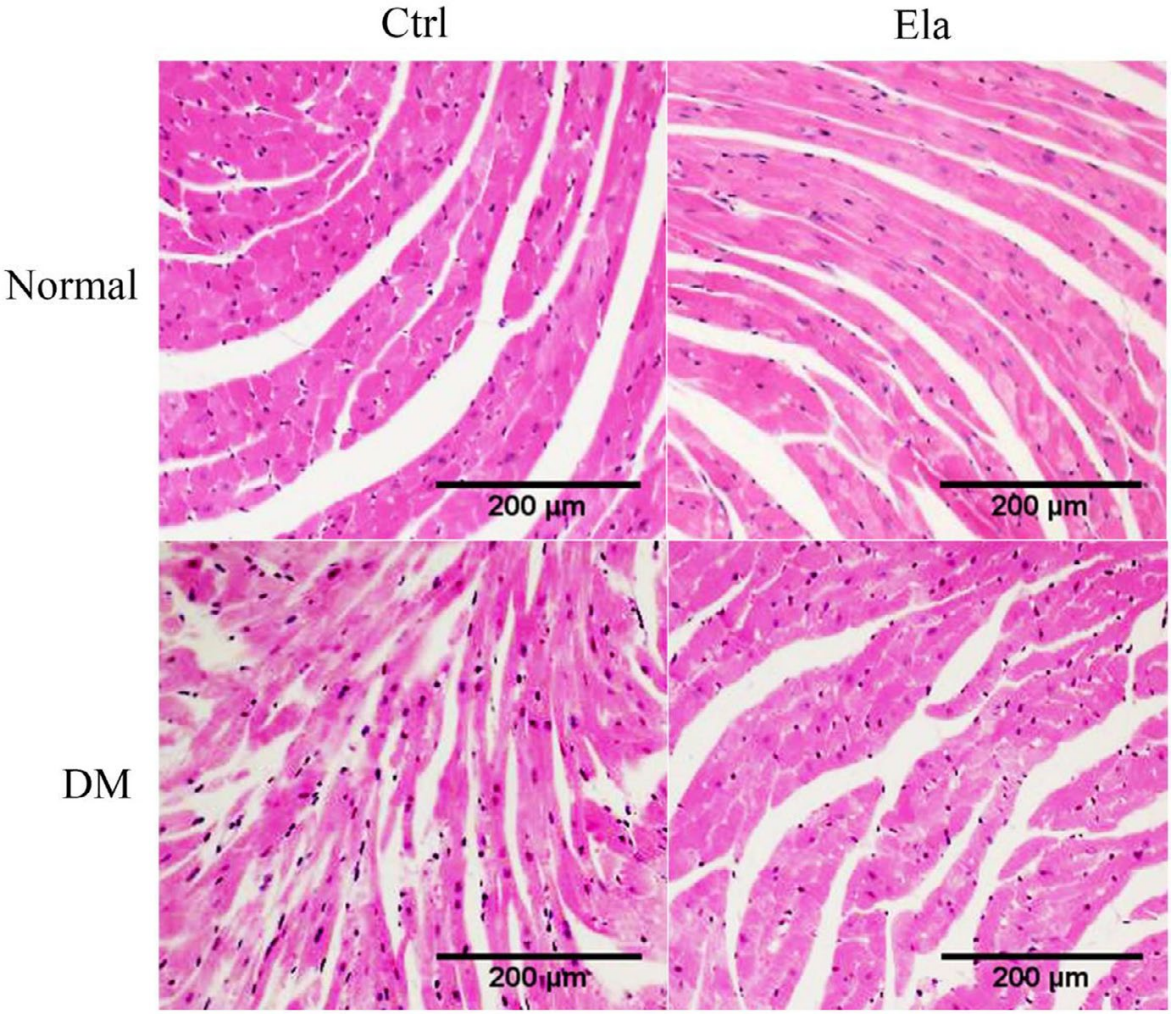
The pathological change of heart tissue in different groups (HE staining, x200). There were noticeable cardiac pathological changes in diabetic mice, such as inflammatory cell infiltration, and myocardial structural changes

We used HE staining to observe the cardiac pathological changes. As expected, cardiac pathological changes (ie inflammatory cell infiltration, and myocardial structure destruction) were widely visible in diabetic mice histology. Elabela treatment alleviated these pathological changes in diabetic mice (Figure 3).

**Figure 4:**
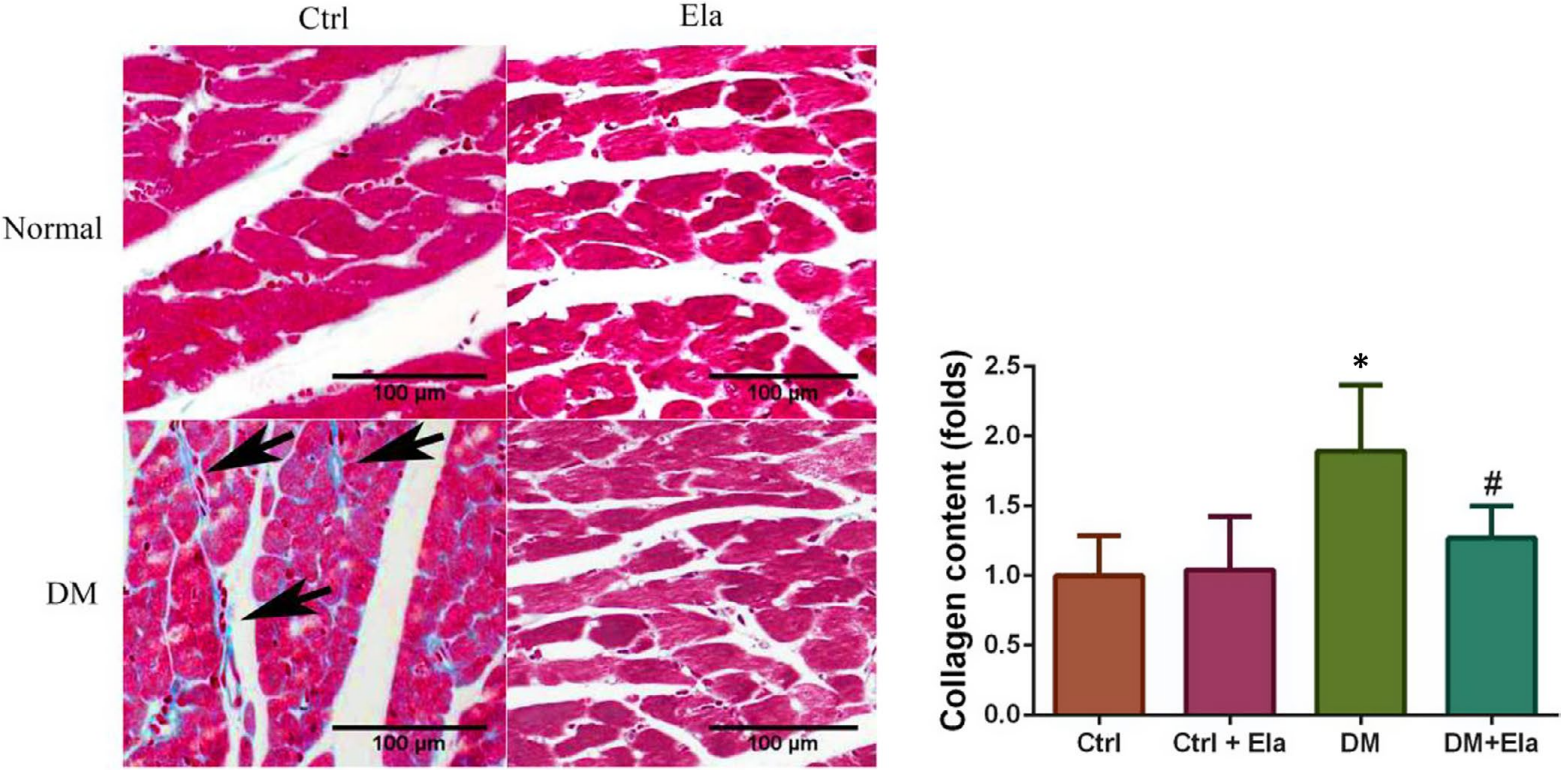
The interstitial collagen accumulation in cardiac tissue of different groups (Masson staining, x400). n = 8. *P < 0.05 DM group vs Ctrl group, ^#^ *P* < 0.05 DM + Ela group vs DM group. DM, diabetes mellitus

We also used Masson staining to evaluate interstitial collagen accumulation in cardiac tissue (Figure 4). The DM group showed increased collagen accumulation when compared to control groups while the DM + Ela group showed markedly less collagen compared to the DM group.

### 3.4 Effect of Elabela on diabetes‐induced cardiac oxidative stress and inflammation

**Figure 5:**
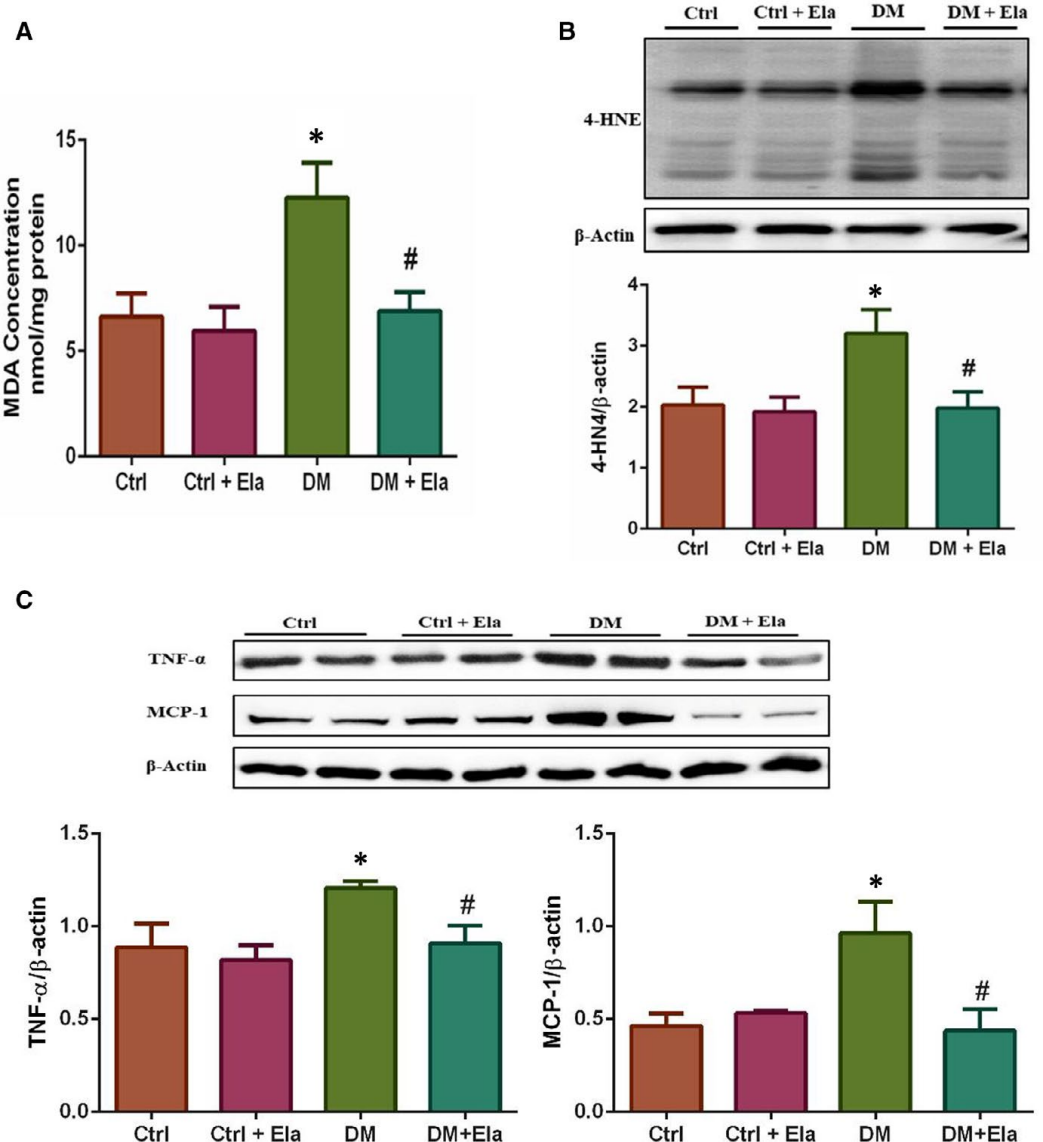
Effect of Elabela on diabetes‐induced cardiac oxidative stress and inflammation. A, MDA was expressed as nmol/mg protein. B, 4‐HNE was detected by Western blot. C, Inflammatory factors TNF‐α and MCP‐1 were detected by Western blot. n = 8. *P < 0.05 DM group vs Ctrl group, ^#^ *P* < 0.05 DM + Ela group vs DM group. DM, diabetes mellitus

We selected MDA and 4‐HNE as oxidative stress indicators, and used the thiobarbituric acid process and Western blot to measure their expressions respectively (Figure 5A,B). The data showed that the concentration of MDA in the DM group's cardiac tissue was drastically increased when compared to the Ctrl group, which was lowered in DM + Ela group (Figure 5A). Similarly, a significantly increased expression of 4‐HNE in the DM group was observed, and the DM + Ela group also showed lower expression (Figure 5B).

We used Western blot to measure the expression of inflammatory factors such as TNF‐α and MCP‐1 (Figure 5C). The Western blot result demonstrated that TNF‐α and MCP‐1 expression levels have distinctly increased in DM mice, while the DM + Ela group had expression levels similar to the Ctrl group.

### 3.5 Effect of Elabela on diabetes‐induced cardiac apoptosis

**Figure 6:**
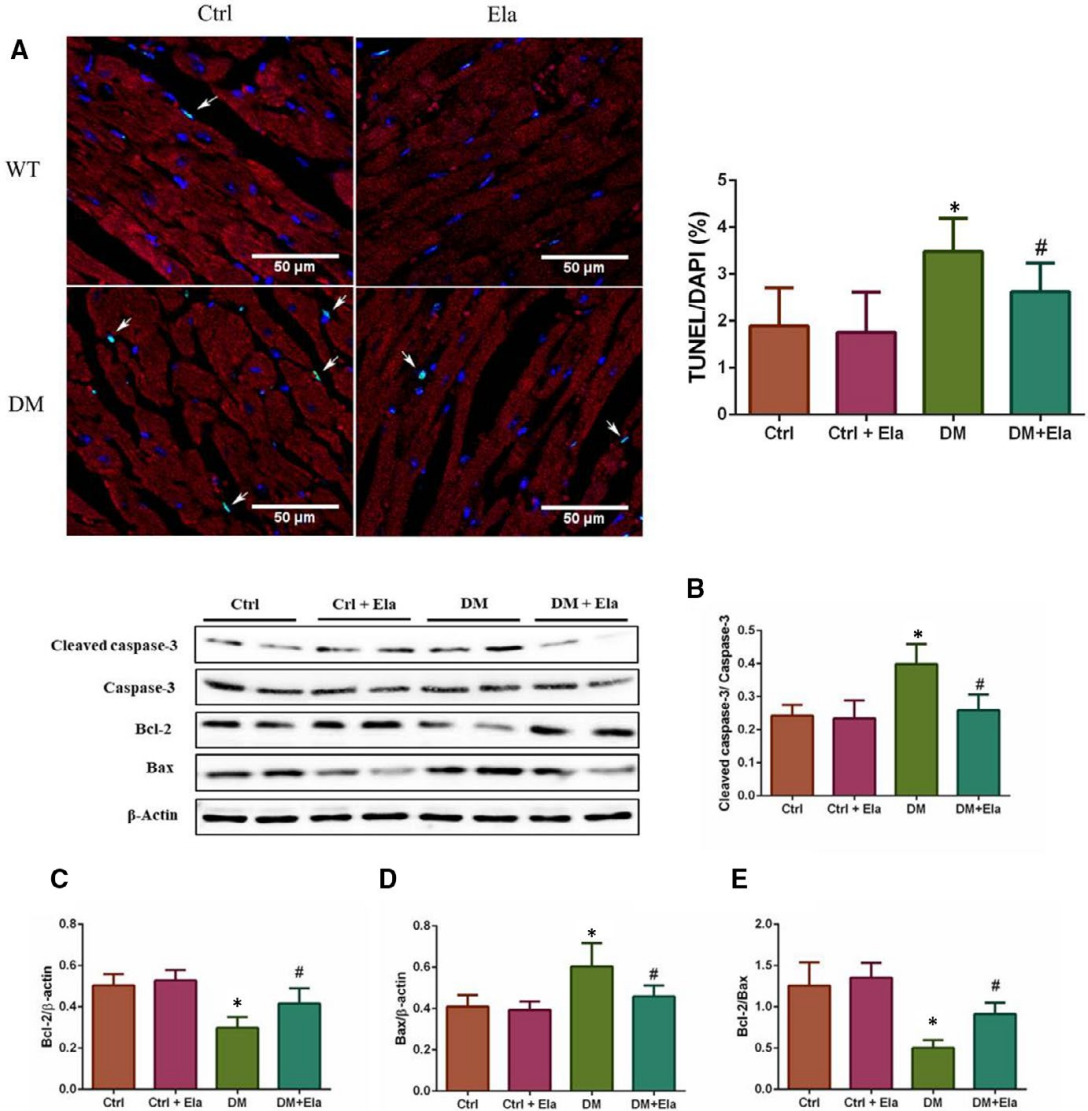
Effect of Elabela on cardiac tissue apoptosis in different groups. A, TUNEL assay was performed to evaluate apoptosis in cardiac tissue (green, X600). (cardiomyocytes were marked by sarcomeric α‐actinin staining in red, and nuclei were counterstained with DAPI). B, The apoptosis‐regulated proteins Bax, Bcl‐2 and cleaved caspase‐3 were detected by Western blot. n = 8. *P < 0.05 DM group vs Ctrl group, ^#^ *P* < 0.05 DM + Ela group vs DM group. DM, diabetes mellitus

We used TUNEL assay to evaluate the apoptosis of myocardial and interstitial cells (cardiomyocytes were marked by sarcomeric α‐actinin staining in red, nuclei were counterstained with DAPI) (Figure 6A), and Western blot to detect the apoptosis‐regulated protein expressions, such as cleaved caspase‐3, total caspase‐3, Bax, and Bcl‐2 (Figure 6B‐E). The result showed that the TUNEL assay‐positive cells are predominately seen in the DM group's cardiac interstitial tissue. The DM + Ela group had fewer diabetic‐induced cell apoptosis. To verify this result, we also examined the expression of cleaved caspase‐3, total caspase‐3, Bax, and Bcl‐2 by Western blot. The ratio of cleaved caspase‐3/ total caspase‐3 and expression of Bax were consistent with the TUNEL results. However, the expression of Bcl‐2 was opposite. We also calculated the Bcl‐2 and Bax ratio, the result also showed an elevated Bcl‐2 and Bax ratio in DM + Ela groups compared to DM groups.

### 3.6 Elabela can up‐regulate the expression of SIRT3 and its downstream antioxidative proteins

**Figure 7:**
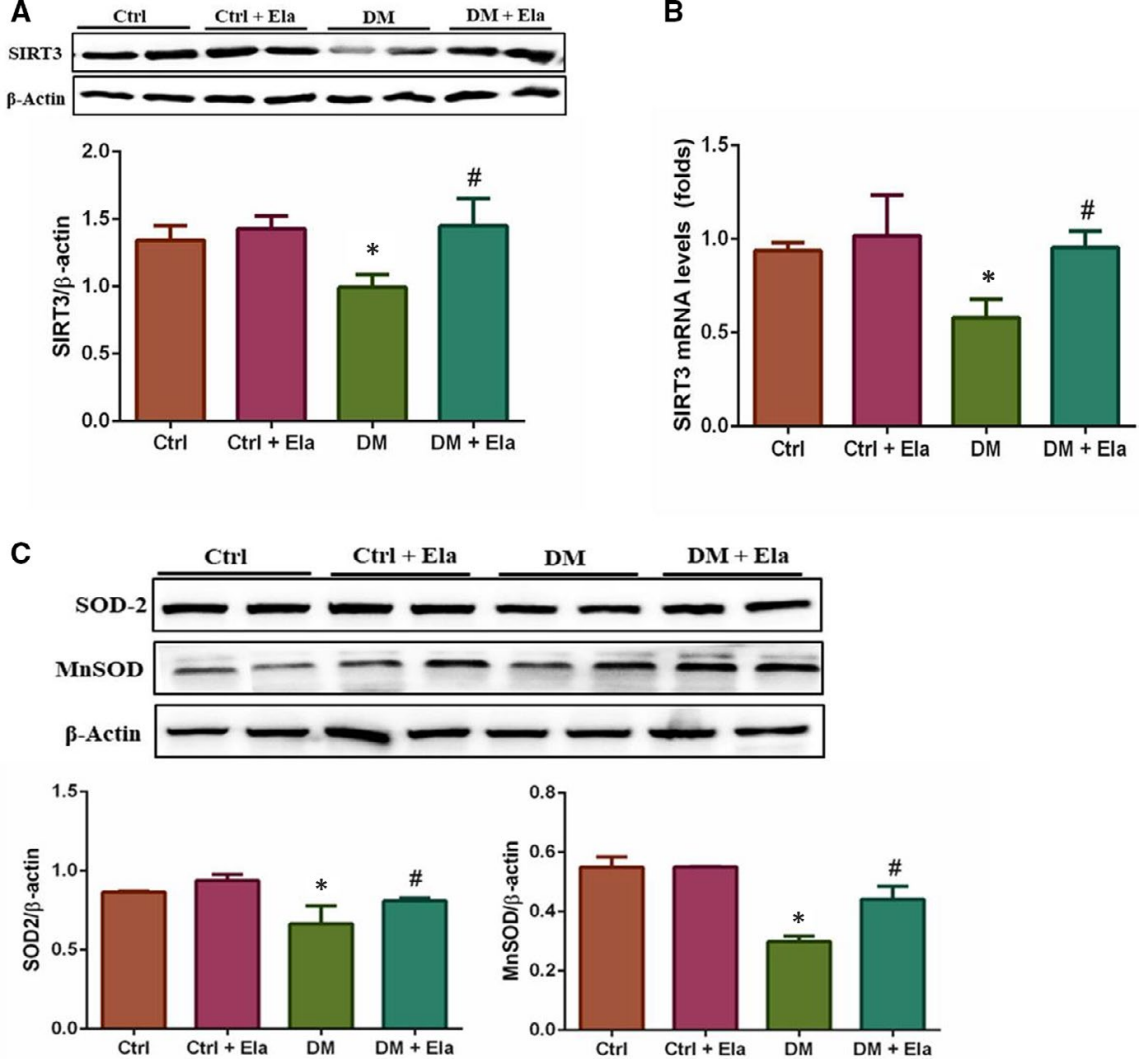
Effect of Elabela on the expressions of SIRT3 and its downstream antioxidative proteins in different groups. The expression of SIRT3 was analysed at the protein level via Western blot (A) and the mRNA level via qPCR (B). Its downstream antioxidative protein SOD‐2 and MnSOD were analysed by Western blot (C). n = 8. *P < 0.05 DM group vs Ctrl group, ^#^ *P* < 0.05 DM + Ela group vs DM group. DM, diabetes mellitus

SIRT3, a key downstream factor of APJ system, has been shown to have an important role in protecting cardiomyocytes from oxidative‐stress‐mediated cell death. Therefore, we used Western blot and RT‐PCR to evaluate the expression of SIRT3 on the protein level (Figure 7A) and transcriptional level (Figure 7B), respectively. The results showed that the expressions of the SIRT3 protein and mRNA in DM mice were lower than in Ctrl ones. The levels of SIRT3 protein and mRNA in the DM + Ela group improved significantly. In addition, we also examined the expressions of SOD‐2 and MnSOD, which were downstream antioxidative proteins (Figure 7C). The results were consistent with SIRT3’s expression levels.

### 3.7 Elabela regulates SIRT3‐induced Foxo3a deacetylation to protect DCM

**Figure 8:**
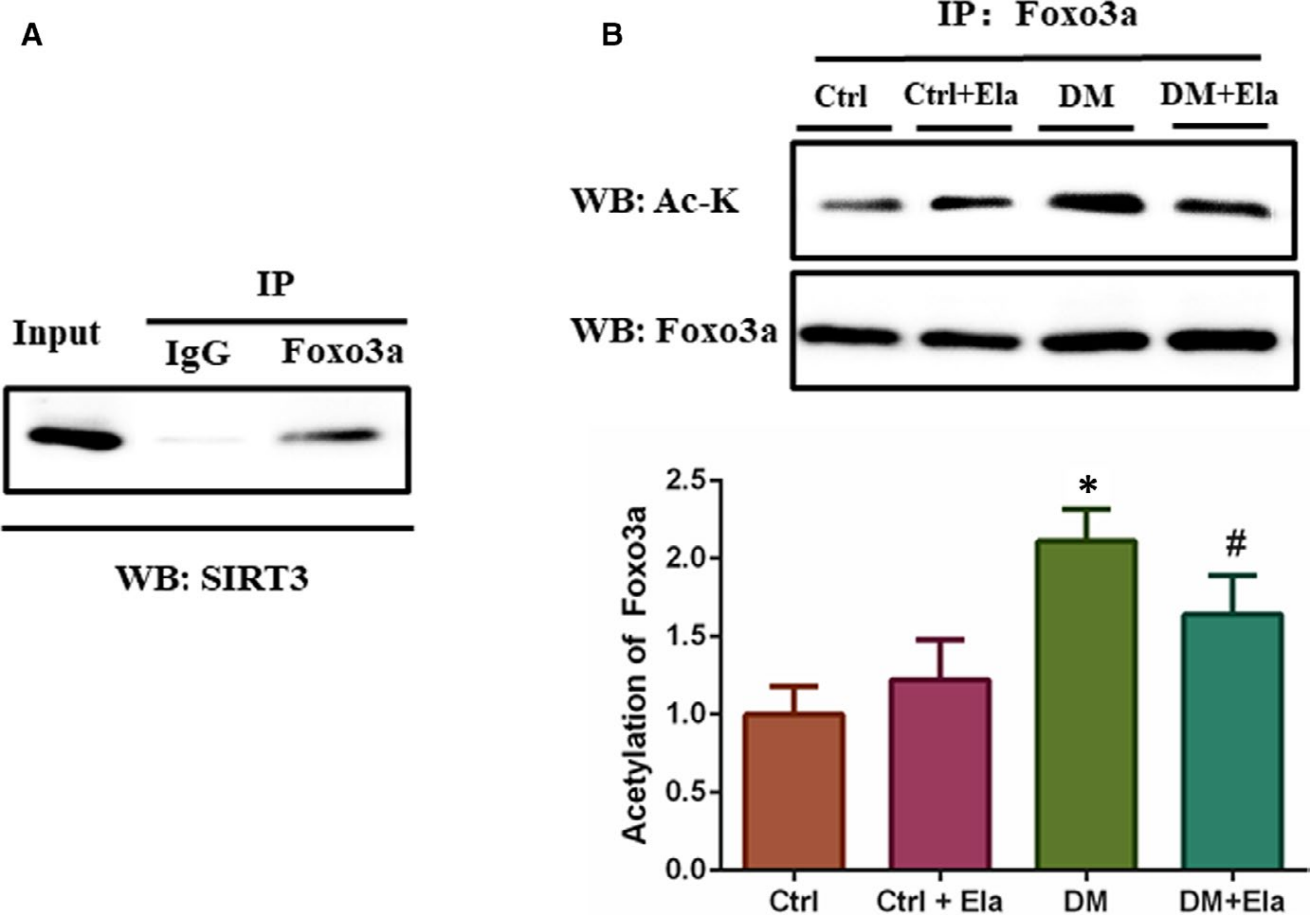
SIRT3’s interaction with Foxo3a, and the acetylation level of Foxo3a in different groups. A, Foxo3a binds to SIRT3 in myocardial cells. Endogenous Foxo3a in myocardial cells was immunoprecipitated using a Foxo3a antibody and analysed by Western blotting using an antibody against SIRT3. B, Foxo3a was immunopurified and probed by acetyl lysine antibody (Ac‐K). n = 8. *P < 0.05 DM group vs Ctrl group, ^#^ *P* < 0.05 DM + Ela group vs DM group. DM, diabetes mellitus

Earlier research showed that diabetic‐induced ROS stimulated SIRT3 expression and that SIRT3 could promote deacetylation‐dependent nuclear entrance of Foxo3a protein.^20^ To verify this hypothesis, we first used Immunoprecipitation assay to examine the ability of SIRT3 to bind to Foxo3a. As shown in Figure 8A, SIRT3 and Foxo3a were able to bind to each other in vivo. Subsequently, we detected the acetylation level of Foxo3a in different groups (Figure 8B). The results showed that the level of Foxo3a acetylation was the highest in the DM group. We observed that Foxo3a acetylation level could evidently be depressed by Elabela treatment as seen in the DM + Ela group. These results were consistent with SIRT3’s expression levels.

## 4 DISCUSSION

Our data, for the first time, demonstrated that the new synthetic peptide Elabela attenuates diabetic‐induced myocardial injury and promotes the expression of SIRT3. Our study showed that diabetes‐induced cardiac oxidative stress, inflammation, fibrosis, and apoptosis can be reduced via the Elabela treatment. We also found that Elabela alleviates DCM via up‐regulation of SIRT3 expression and Foxo3a deacetylation.

Oxidative stress is considered to be one of the underlying mechanisms of diabetic complications. Apelin, a bioactive peptide isolated from bovine gastric extract, is an endogenous ligand of the human G‐protein‐coupled receptor APJ. Apelin/APJ is expressed in multiple tissues, including vascular endothelial cells and myocardium.^23^ There are strong connections between Apelin/APJ and oxidative stress. There is evidence showing that Apelin has a close association with diabetic complications.^13^, ^24^ SIRT3 is localized primarily in the mitochondria. As a major mitochondrial deacetylase, it plays a pivotal role in deacetylating and modifying activities of several mitochondrial enzymes.^25^ The mitochondrial electron transport chain is the main source of ROS, and SIRT3 can enhance mitochondria's ROS coping capacity by multiple factors.^26^ Recent studies indicated that the protective effect of Apelin/APJ system may depend on SIRT3 and its downstream factors.^27^, ^28^, ^29^ A research showed that overexpression of Apelin significantly attenuated high glucose‐induced ROS and improved the expression of SIRT3, while Apelin's protection against high glucose‐induced ROS formation was diminished when SIRT3 was knocked out.^19^ Hou et al also demonstrated that SIRT3 is essential for Apelin‐induced angiogenesis in post‐myocardial infarction of diabetes.^27^ In addition, Apelin/SIRT3 signaling has been demonstrated to improve mitochondrial dysfunction in DCM,^29^ and SIRT3 deficiency promotes cardiac dysfunction, due to defective trans‐mitochondrial cristae alignment and impaired mitochondrial bioenergetics.^30^

In our study, Elabela, the new endogenous agonist of APJ, demonstrated the similar functions in DCM as Apelin. We used Elabela to treat diabetic mice, which significantly attenuating the factor expressions of oxidative stress, inflammation, fibrosis, and apoptosis to support the notion that Elabela has protective effect against DCM. Moreover, in this study, we verified that Elabela therapy improves cardiac function via modulation of SIRT3 in diabetic mice by examining the expression of SIRT3, shown by the fact.that SIRT3 expression was readily down‐regulated in DM group, which could be increased by the Elabela treatment. This suggests that Elabela enhances cardiac function probably via up‐regulating SIRT3 in DCM.

The SIRT3 substrate, Foxo3a, is a Forkhead transcription factor that mediates multiple genes expressions, governing several different cellular processes. FOXO3a activity is regulated by post‐translational modifications that drive its shuttling between different cellular compartments, thereby determining its activation (ie nucleus and mitochondria) or inactivation (ie cytoplasm).^31^ Key superoxide scavengers, MnSOD and SOD‐2, can reduce superoxide production and protect against oxidative stress. While SIRT3 can sequester Foxo3a via deacetylation in the nucleus to increase the transcription of MnSOD, SOD‐2, and other key genes involved in antioxidation.^32^, ^33^, ^34^ There were evidence depicting that SIRT3‐mediated deacetylation of Foxo3a reduced levels of ROS by up‐regulating antioxidant enzymes.^35^, ^36^ Therefore, we first examined the ability of SIRT3 to bind to Foxo3a. As shown in Figure 8A, SIRT3 and Foxo3a were able to bind to each other in vivo. To determine whether Elabela can directly reduce acetylation of Foxo3a, we examined the deacetylation level of Foxo3a in different groups. We observed that diabetes significantly increased the level of acetylated Foxo3a while diabetes treated with Elabela decreased acetylated Foxo3a expression. Furthermore, we determined the MnSOD and SOD‐2 expression levels. We also found that in the DM group the expressions of MnSOD and SOD‐2 were greatly suppressed; however, they could be alleviated by Elabela treatments. These results were consistent with Foxo3a deacetylation levels. This suggests that the protective effects of Elabela may be related to SIRT3‐mediated deacetylation of Foxo3a.

In summary, our findings reveal that Elabela has protective effects against DCM, which may depend on SIRT3‐mediated deacetylation of Foxo3a, promoting antioxidant factor expressions. This hints that Elabela may be used as a novel therapy in treating DCM‐afflicted patients as our findings provide new strategies to prevent and improve DCM‐related pathological changes.

## CONFLICT OF INTEREST

No conflicts of interest.

## AUTHOR CONTRIBUTION

**Cheng Li:** Conceptualization (lead); Data curation (lead); Formal analysis (lead); Investigation (lead); Methodology (lead); Software (lead); Visualization (lead); Writing‐original draft (lead); Writing‐review & editing (lead). **Xiao Miao:** Data curation (equal); Formal analysis (equal). **Shudong Wang:** Funding acquisition (supporting); Resources (supporting); Writing‐review & editing (equal). **Yucheng Liu:** Writing‐review & editing (lead). **jian sun:** Funding acquisition (equal); Resources (equal); Supervision (equal). **quan liu:** Funding acquisition (equal); Supervision (equal). **Lu Cai:** Conceptualization (lead); Supervision (lead); Validation (lead); Writing‐review & editing (lead). **yonggang wang:** Conceptualization (lead); Data curation (lead); Funding acquisition (lead); Resources (lead); Writing‐review & editing (lead).

## Data Availability

All the data used to support the findings of this study are included within the article.
